# Combined Phase 1/2a Initial Clinical Safety Trials and Proof‐of‐Concept Assessment of a Novel Antimicrobial Peptide KSL‐W Anti‐Plaque Chewing Gum

**DOI:** 10.1002/cre2.943

**Published:** 2024-10-28

**Authors:** J. Brett Ryan, Brian J. Kirkwood, Kai P. Leung

**Affiliations:** ^1^ US Air Force Dental Research and Consultation Service San Antonio Texas USA; ^2^ Clinical Technology Integration US Army Institute of Surgical Research San Antonio Texas USA; ^3^ Combat Wound Care Group US Army Institute of Surgical Research San Antonio Texas USA

**Keywords:** antimicrobial peptide, dental caries, KSL‐W, oral health, plaque

## Abstract

**Objectives:**

The effective control of dental plaque is crucial for oral health, given that pathogenic bacteria in plaque are the primary cause of dental caries. Current antimicrobial agents, although effective, disrupt the oral microbiome and lead to oral dysbiosis, hindering efforts to curb dental caries. Novel antimicrobial peptides offer a promising solution due to their selective bactericidal activity against cariogenic bacteria. This study explores the initial safety and efficacy of KSL‐W formulated into chewing gum through a Phase 1 and 2a clinical trial.

**Methods:**

The combined trial, approved by the FDA, follows a double‐blind, randomized, placebo‐controlled design. Phase 1 assessed safety with single doses (2−100 mg), whereas Phase 2a explored both safety and proof of concept in reducing oral bacteria with multiple doses (4−75 mg). Besides adverse events (Phase 1), outcome measures included whole‐mouth plaque and gingival index scores and bleeding on probing (Phase 2a).

**Results:**

KSL‐W demonstrated safety in both phases, with no severe adverse events. The proof‐of‐concept analysis revealed a decrease in plaque and gingival inflammation, particularly at doses ≥ 20 mg. The 30 mg dose appeared to yield optimal effects without any adverse reactions in subjects.

**Conclusions:**

Results from this study indicate that KSL‐W is safe for use in humans and provides initial evidence of its potential efficacy in reducing plaque and gingival inflammation. Further research is essential to determine optimal usage and ultimate safety, and to assess its potential in diverse populations.

**Trial Registration:**

The trial is registered with the FDA (Trial Registration Number: NCT01877421). The clinical trials were registered in the clinicaltrials.gov database under the title “Safety and Tolerability of Antiplaque Chewing Gum in a Gingivitis Population” and the identifier number is NCT01877421. The URL for accessing the study in clinicaltrials.gov is https://clinicaltrials.gov/study/NCT01877421?intr=Antiplaque%20chewing&rank=1.

## 1 Introduction

The effective control of dental plaque is critical to maintaining good oral health because the uncontrolled growth of pathogenic bacteria that reside within dental plaque is the primary etiology of dental caries (i.e., dental “decay”), the most common untreated non‐communicable disease in the world (Marsh 2010; Henshaw, Garcia, and Weintraub 2018; WHO 2023). Dental plaque biofilms are also a precursor to the development of periodontal diseases. The primary means of managing dental plaque is through routine oral hygiene (i.e., daily brushing and flossing) that mechanically disrupts the establishment of cariogenic bacteria on intraoral hard and soft tissue (Haps et al. 2008; Marsh 2010). In certain scenarios, where routine oral hygiene is not enough to maintain good oral health, dental providers often recommend or prescribe antibacterial agents to patients as an adjunctive. Although these additional therapeutics are effective in eliminating the bacteria that cause caries, their broad‐spectrum affects kill off beneficial oral flora as well (Marsh 2010). This indiscriminate eradication of oral bacteria often favors the growth of pathogenic bacteria over commensal bacteria during the recolonization of intraoral hard and soft tissue surfaces and can lead to the disruption of the oral microbiome, akin to the negative effects that systemic antibiotics have on the gut microbiome (Rosier, Marsh, and Mira 2018). This disruption, referred to as oral dysbiosis, has been linked to higher rates of dental caries, periodontal disease (gum disease), and oral cancer, along with common systemic diseases such as cardiovascular disease and diabetes (Georges, Do, and Seleem 2022; Maier 2023). This means that the use of currently available intraoral antimicrobial agents may not only be hindering efforts by oral healthcare providers to make mouths healthier but they might also be harmful to overall wellness (Ghosh et al. 2018; Rosier, Marsh, and Mira 2018; Maier 2023).

A potential solution for this is the use of antimicrobial peptides that have shown tremendous promise in the treatment of a variety of conditions where traditional chemotherapeutic agents have encountered resistance, failed, or led to long‐term damaging effects (Kumar et al. 2019; Li et al. 2023). Antimicrobial peptides are small polypeptides of 12−50 amino acids that are naturally produced by the immune systems of a variety of organisms, mammals included, that regulate and aid the body's response to various types of pathogens, including fungi, bacteria, and viruses (Li et al. 2023). Generally, antimicrobial peptides are not present in high enough concentrations to combat severe or widespread challenges to the human immune system, and this is especially true in saliva, where they are readily diluted and easily degraded by enzymes present in the mouth (Wang, Shen, and Haapasalo 2017; Haney, Straus, and Hancock 2019). This means that any antimicrobial peptide intended to be used as an adjunctive to strengthen the antimicrobial defense intraorally would need to be easily deliverable and usable, highly concentrated, and amenable to a sustained release.

KSL‐W, a synthetic novel antimicrobial peptide developed by Leung et al. (2009), can meet these three requirements when formulated into chewing gum. In vitro, KSL‐W has demonstrated selective bactericidal activity against known cariogenic bacteria (e.g., *Streptococcus mutans* and *Lactobacillus acidophilus*) and early colonizers (e.g., *Actinomyces naeslundii*) associated with periodontitis (Leung et al. 2009; Semlali et al. 2011). It is resistant to enzymatic degradation in the mouth for up to 60 min and also shows antibacterial activity within minutes after simulated chewing releases it from a delivery vehicle (Faraj et al. 2007; Leung et al. 2009). KSL‐W has sustained antimicrobial action when exposed to common intraoral enzymes and conditions as well, a critical feature for any ingested agent (Faraj et al. 2007; Al‐Ghananeem et al. 2017). Studies also indicate that KSL‐W is absorbed and released by hydroxyapatite, a key mineral component of teeth (Leung et al. 2009). Additionally, delivering the peptide with gum has the added benefit of increased salivary flow, a very important factor for good oral health that is stimulated by mastication (i.e., chewing).

Though these in vitro studies have demonstrated the tremendous potential of KSL‐W as an intraoral antimicrobial agent, no research has been conducted to establish whether KSL‐W is safe in humans and or even whether it would work in vivo as theorized. Therefore, we set out to conduct a combined Phase 1/2a clinical trial to establish an initial safety profile and potential efficacy of the antimicrobial peptide KSL‐W added to chewing gum. Positive results from this study would not only mark a significant step toward developing an over‐the‐counter (OTC) anti‐plaque chewing gum but would also have broad implications for oral health worldwide, especially in vulnerable populations with limited access to oral care.

## 2 Methods

The Indiana University Institution Review Board and the Office of Human Research Oversight at the US Army Medical Research and Development Command granted approval for this study. The protocol number for the study is A1305011355 (Indiana University Institution Review Board). This study was also approved and registered with the Food and Drug Administration, trial registration number (NCT01877421) under IND 77532 S‐11‐14. Appropriate informed consent was received from all participants in this study according to the established protocol.

### 2.1 Trial Design

This was a combined Phase 1/2a placebo‐controlled, double‐blind, randomized, dose‐escalation pilot study to evaluate both the safety of KSL‐W and the proof of concept for its use in an Antiplaque Chewing Gum (APCG) formulation. Doses administered throughout both phases consisted of 1 piece of gum, and subjects chewed the gum for approximately 20 min under supervision. Subjects were instructed to chew the gum on each side of the mouth for the first 5 min, alternating sides approximately every 15 s. After this period, the subjects were instructed to continue chewing as they normally would for another 15 min. In both phases, a Data Review Committee (DRC) was put in place to review the occurrence of systemic adverse events (AEs), oral soft tissue (OST) AEs, and oral hard tissue (OHT) AEs, as indicated by changes from baseline that could be attributable to the APCG. For both phases, group size was determined based on a priori sample size analysis, and group assignment was randomized.

To evaluate the safety, Phase 1 consisted of nine sequential groups (Groups 1a−9a) that received a single dose of KSL‐W in the APCG that started at 2 mg and went to 100 mg, as well as a paired placebo group for each dosing group (see Table 1). Once it was determined by the DRC that no AEs had occurred, the next group of participants was administered the next higher dose. Phase 1 subjects received a single oral dose of 2, 4, 6, 10, 20, 30, 50, 75, or 100 mg of KSL‐W chewing gum per assigned group (see Table 1).

**Table 1:** Treatment groups for study phases and dosage of KSL‐W antimicrobial peptide.

		No of Subjects (*n*)		
		Total	Active treatment	Placebo
Phase 1 dose of KSL‐W				
1a	2 mg	3	2	1
2a	4 mg	6	4	2
3a	6 mg	6	4	2
4a	10 mg	6	4	2
5a	20 mg	10	7	3
6a	30 mg	10	7	3
7a	50 mg	10	7	3
8a	75 mg	10	7	3
9a	100 mg	10	7	3
Phase 2a				
1b	4 mg	6	4	2
2b	6 mg	10	7	3
3b	10 ng	10	7	2
4b	20 mg	10	6	3
5b	30 mg	10	7	3
6b	50 mg	10	7	3
7b	75 mg	10	7	3

Phase 2a continued to assess the safety of the APCG along with an evaluation of the potential efficacy of the gum to lower plaque biofilm in vivo. This phase consisted of seven dosing groups (Groups 1b−7b) that received different amounts of KSL‐W in the APCG in an escalating fashion identical to Phase 1. Subjects received multiple oral doses of 4, 6, 10, 20, 30, 50, or 75 mg of KSL‐W for 28 days on a pre‐determined schedule; one dose was administered on study days 0−6, two doses on study days 7−20, and three doses on study days 21−28 (see Table 2). Investigators did not initiate testing for participants in the next higher dose group until the DRC confirmed the absence of adverse reactions.

**Table 2:** Treatment groups and dose timing for study phases.

Study phase	Phase 1/2a	Phase 2a			
Study day	0	1−6	7−13	14−20	21−27
Unspecified time	X				
After breakfast		X	X	X	X
After lunch			X	X	X
After dinner					X

To test the proof of concept in Phase 2a that the APCG could reduce intraoral bacteria, we assessed three indicators known to be associated with excessive bacteria growth: supragingival plaque, gingivitis, and bleeding on probing (BOP). Supragingival plaque was assessed using the Turesky Modification of the Quigley–Hein Plaque Index (Turesky, Gilmore, and Glickman 1970), gingivitis using the Modified Gingival Index (Lobene et al. 1986), and the percent of BOP using the methods described by Ainamo and Bay (1975); all measures were calculated as whole‐mouth scores, meaning that they were combined scores for all quadrants. Participants were supervised for chewing during the regular work week (Monday to Friday), and unsupervised on the weekends and dinner time. For supervised chews, all subjects were instructed to chew the gum under the direction of the principal investigator or designee using a standardized 20 min chewing time. The maximum dose in a 24 h period was three 75 mg chewing gum tablets.

### 2.2 Criteria for Inclusion and Exclusion

For Phase 1, males and females between the ages of 18 and 64 years (inclusive) who self‐reported that they had good systemic health with a minimum of 16 natural teeth were included. For Phase 2a, subjects needed to meet the Phase 1 inclusion criteria but also had to have mild to moderate gingivitis with visible plaque deposits. Finally, a total of 135 subjects (71 in Phase 1 and 64 in Phase 2a) were enrolled.

### 2.3 Study Subjects

**Figure 1:**
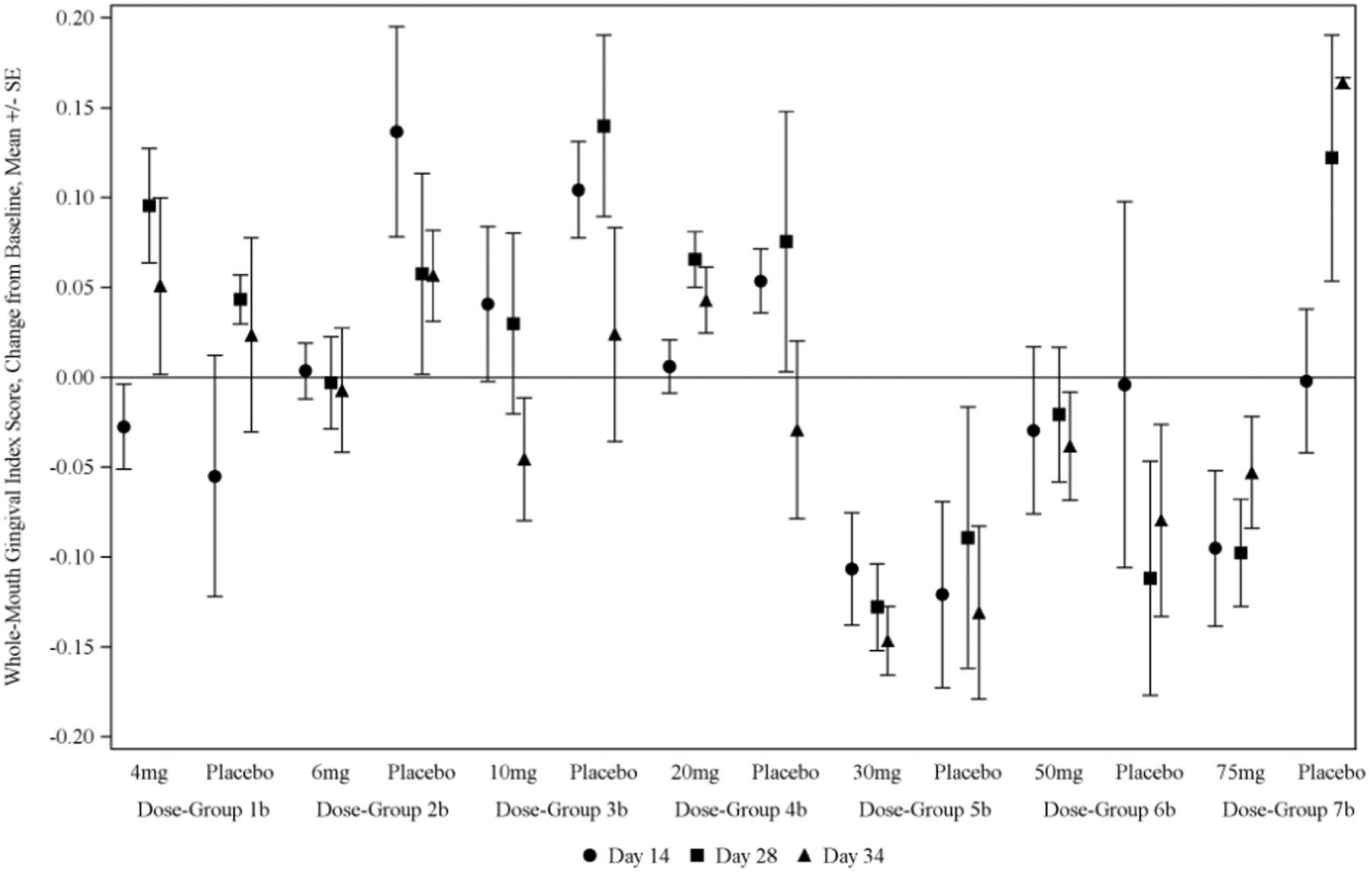
Whole‐mouth gingival index score change (mean change ± standard error) from baseline in the Phase 2a study.

**Figure 2:**
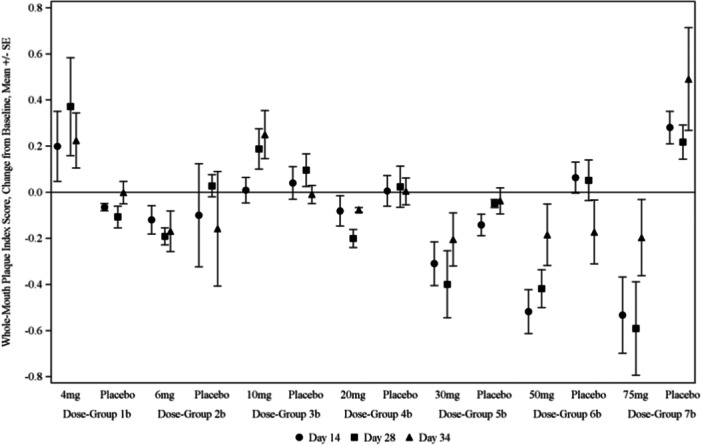
Whole‐mouth plaque index score change (mean change ± standard error) from baseline Phase 2a.

In Phase 1, a total of 84 subjects were screened, 13 subjects were disqualified, and the remaining 71 subjects completed the study after being randomly assigned to the various dosing groups. Seventy‐nine subjects were initially assessed for Phase 2a, 15 subjects were disqualified, and the remaining 64 participants were randomized to active and placebo gum groups (see Supporting Information S1: Tables 1 and 2). For complete subject disposition, refer to Supporting Information S1: Figure 1 (Phase 1) and Figure 2 (Phase 2a).

### 2.4 Blinding

No blinding occurred in Phase 1; the goal of this phase was to assess the single‐dose safety of KSL‐W. Phase 2a was double‐blinded, and the placebo gum and gum containing KSL‐W were provided without labels to the research team in charge of distribution to clinicians and patients; therefore, neither the clinicians nor the staff interacting with patients knew which sample contained the active ingredient or placebo.

### 2.5 Statistical Analysis

All AEs were recorded and presented as by‐subject data listings and treatment‐emergent AEs (an AE that occurred after administration of the first dose of study treatment or any medical or dental condition present at baseline that worsened after administration of the first dose of study treatment) were tabulated at the subject level. Summaries for these safety parameters included changes from baseline to each post‐baseline evaluation time point, and complete results are available in the trial technical report.

The proof‐of‐concept endpoints for PI, MGI, and BOP were initially evaluated by comparing changes from baseline with paired *t*‐tests at Days 14, 28, and 34 for all groups, including placebo. To assess differences between the various doses of KSL‐W administered at 14, 21, and 34 days, an ANOVA with post hoc adjustments was performed by calculating the mean difference between the whole‐mouth scores for the proof‐of‐concept endpoint between those who received the placebo and APCG for each dose group. Finally, to account for differences in baseline scores, as well as all subjects who received a placebo, an ANCOVA was performed to assess for differences between KSL‐W dose groups against all placebo results pooled together.

## 3 Outcomes

### 3.1 Safety

The Phase 1 single‐dose escalation (2−100 mg KSL‐W) and Phase 2a multi‐dose escalation (4−75 mg KSL‐W) were completed without any adverse safety events that required a subject to withdraw, as verified by the DRC, study site PI, and qualified study physician. In Phase 1, nine subjects experienced 10 AEs (four placebo, six active); however, all were classified as mild and did not require investigators to cease the study (see Supporting Information S1: Table 1). Thirty‐one of the 64 (48.4%) Phase 2a subjects experienced 54 AEs, 43 in the active group and 11 in the placebo group (see Supporting Information S1: Table 2). As with Phase 1, none of the AEs necessitated halting the investigation; nine (16.7%) were classified as moderate and 45 (83.3%) were classified as mild. Only one moderate event at the 50 mg dose was considered to possibly be product‐related, but a further review by the DRC concluded that it was not. Of the mild events, only four were considered to be related to the product, but only at the 75 mg dose. There were no study‐related laboratory findings or notable vital signs, physical exam findings, or other observations that were clinically significant. Complete results from the trial can be found in Supporting Information S2: Tables 3−5.

### 3.2 Proof of Concept

In Phase 2a, the average whole‐mouth plaque scores and gingival index scores decreased from baseline for groups who received 20 mg of KSL‐W or more at 14, 21, and 34 days (Figure 1), but there was no consistent pattern for BOP (Table 3). Direct comparisons between active and placebo treatments within each dose group did not reveal that KSL‐W improved outcome measures for most proof‐of‐concept endpoints across post‐baseline time points (Table 4). When results were adjusted for subject‐wise baseline scores, those who received 30, 50, or 75 mg of KSL‐W had lower plaque scores at all post‐baseline time points compared with all placebo subjects' PI scores pooled together (Figure 2); also, subjects who received 30 mg of KSL‐W had lower GI scores after 14 days of use (Table 5).

**Table 3:** Paired *t*‐tests comparing the mean baseline scores (Day 0) to selected post‐baseline time points for each dose/placebo group for proof‐of‐concept endpoints.

		Dose group 1b		Dose group 2b		Dose group 3b		Dose group 4b		Dose group 5b		Dose group 6b		Dose group 7b	
		4 mg (*N* = 4)	Placebo (*N* = 2)	6 mg (*N* = 7)	Placebo (*N* = 3)	10 mg (*N* = 7)	Placebo (*N* = 2)	20 mg (*N* = 6)	Placebo (*N* = 3)	30 mg (*N* = 7)	Placebo (*N* = 3)	50 mg (*N* = 7)	Placebo (*N* = 3)	75 mg (*N* = 7)	Placebo (*N* = 3)
		Mean (SE)													
Plaque index	Day 14	0.2 (0.15)	−0.06 (0.02)	−0.12 (0.06)	−0.1 (0.22)	0.01 (0.06)	0.04 (0.07)	−0.08 (0.07)	0.01 (0.07)	**−0.31 (0.09)** *	−0.14 (0.05)	**−0.52 (0.1)** *	0.06 (0.07)	**−0.53 (0.17)** *	0.28 (0.07)
	Day 28	0.37 (0.21)	−0.11 (0.05)	**–0.19 (0.04)** *	0.03 (0.05)	0.19 (0.09)	0.1 (0.07)	**−0.2 (0.04)** *	0.02 (0.09)	**−0.4 (0.15)** *	−0.05 (0.02)	**−0.42 (0.08)** *	0.05 (0.09)	**−0.59 (0.2)** *	0.22 (0.07)
	Day 34	0.22 (0.12)	0 (0.05)	−0.17 (0.09)	−0.16 (0.25)	0.25 (0.1)	−0.01 (0.04)	**−0.08 (0.01)** *	0 (0.06)	−0.21 (0.12)	−0.04 (0.06)	–0.18 (0.13)	−0.17 (0.14)	−0.2 (0.17)	0.49 (0.22)
Gingival index	Day 14	−0.03 (0.02)	−0.06 (0.07)	0 (0.02)	0.14 (0.06)	0.04 (0.04)	0.1 (0.03)	0.01 (0.01)	0.05 (0.02)	**−0.11 (0.03)** *	−0.12 (0.05)	−0.03 (0.05)	0 (0.1)	−0.1 (0.04)	0 (0.04)
	Day 28	0.1 (0.03)	0.04 (0.01)	0 (0.03)	0.06 (0.06)	0.03 (0.05)	0.14 (0.05)	**0.07 (0.02)** *	0.08 (0.07)	**−0.13 (0.02)** *	−0.09 (0.07)	−0.02 (0.04)	−0.11 (0.07)	**−0.1 (0.03)** *	0.12 (0.07)
	Day 34	0.05 (0.05)	0.02 (0.05)	−0.01 (0.03)	0.06 (0.03)	−0.05 (0.03)	0.02 (0.06)	0.04 (0.02)	−0.03 (0.05)	**−0.15 (0.02)** *	−0.13 (0.05)	−0.04 (0.03)	−0.08 (0.05)	−0.05 (0.03)	**0.16 (0)** *
Bleeding on probing	Day 14	−4% (3%)	−8% (5%)	−8% (4%)	5% (4%)	1% (3%)	−11% (5%)	−2% (2%)	−4% (5%)	**−7%(3%)** *	2% (6%)	−6% (5%)	4% (7%)	−6% (%5%)	−2% (3%)
	Day 28	−5% (3%)	−12% (1%)	−4% (3%)	4% (5%)	−2% (2%)	−2% (12%)	**5% (2%)** *	−10% (15%)	−6% (3%)	5% (3%)	−4% (2%)	3% (13%)	1% (6%)	−4% (4%)
	Day 34	−2% (5%)	9% (14%)	−11% (6%)	2% (6%)	1% (3%)	2% (22%)	4% (7%)	−6% (11%)	−8% (3%)	**–7 %(1%)** *	−12% (6%)	−3% (9%)	−2% (4%)	3% (1%)

* Bold values indicate a result with *p* ≤ 0.05.

**Table 4:** ANOVA comparing differences of change in the mean baseline scores (Day 0) to selected post‐baseline time points for each dose/placebo group for proof‐of‐concept endpoints.

		Dose group 1b	Dose group 2b	Dose group 3b	Dose group 4b	Dose group 5b	Dose group 6b	Dose group 7b
		4 mg (*N* = 4)	6 mg (*N* = 7)	10 mg (*N* = 7)	20 mg (*N* = 6)	30 mg (*N* = 7)	50 mg (*N* = 7)	75 mg (*N* = 7)
		Mean difference of active—placebo (SE)						
Plaque index	Day 14	0.02 (0.06)	−0.12 (0.15)	−0.11 (0.12)	−0.01 (0.21)	−0.26 (0.17)	**−0.57 (0.19)** *	**−0.48 (0.17)** *
	Day 28	0.15 (0.13)	−0.27 (0.04)	−0.13 (0.14)	−0.37 (0.16)	−0.5 (0.25)	−0.35 (0.15)	−0.42 (0.31)
	Day 34	0.03 (0.04)	−0.16 (0.16)	−0.03 (0.13)	0.05 (0.06)	−0.35 (0.16)	−0.07 (0.28)	−0.38 (0.29)
Gingival index	Day 14	−0.01 (0.04)	**−0.12 (0.04)** *	‐0.07 (0.09)	−0.05 (0.03)	0.01 (0.06)	−0.06 (0.07)	0.01 (0.05)
	Day 28	0.06 (0.06)	−0.05 (0.05)	−0.12 (0.1)	−0.01 (0.06)	−0.05 (0.05)	0.07 (0.08)	**−0.11 (0.04)** *
	Day 34	0.01 (0.1)	−0.06 (0.06)	−0.08 (0.07)	0.07 (0.05)	−0.03 (0.03)	0 (0.04)	−0.13 (0.05)
Bleeding on probing	Day 14	3% (6%)	−10% (7%)	**16% (6%)** *	0% (%2%)	−7% (6%)	**−12 %(3%)** *	−1% (4%)
	Day 28	9% (6%)	−6% (6%)	−4% (8%)	10% (10%)	−9% (5%)	−9% (9%)	8% (7%)
	Day 34	−16% (0%)	−5% (0%)	−6% (0%)	6% (0%)	2% (0%)	−13% (0%)	−2% (0%)

* Bold values indicate a result with *p* ≤ 0.05.

**Table 5:** ANCOVA comparing differences of change in pooled baseline scores (Day 0) to selected post‐baseline time points for each dose/placebo group for proof‐of‐concept endpoints.

		Dose group 2b	Dose group 3b	Dose group 4b	Dose group 5b	Dose group 6b	Dose group 7b
		6 mg (*N* = 7)	10 mg (*N* = 7)	20 mg (*N* = 6)	30 mg (*N* = 7)	50 mg (*N* = 7)	75 mg (*N* = 7)
		Mean difference of active versus pooled placebo (SE)					
Plaque index	Day 14	**−0.17 (0.07)** *	−0.11 (0.07)	−0.1 (0.08)	**−0.35 (0.08)** *	**−0.58 (0.09)** *	**–0.73 (0.1)** *
	Day 28	**−0.26 (0.04)** *	0.02 (0.06)	**−0.25 (0.05)** *	**−0.46 (0.09)** *	**−0.47 (0.07)** *	**−0.82 (0.12)** *
	Day 34	**−0.24 (0.08)** *	0 (0.1)	−0.11 (0.09)	**−0.28 (0.1)** *	**−0.3 (0.12)** *	**−0.45 (0.11)** *
Gingival index	Day 14	0 (0.05)	0.03 (0.05)	−0.01 (0.06)	**−0.12 (0.05)** *	−0.07 (0.06)	**−0.13 (0.06)** *
	Day 28	−0.01 (0.05)	0.01 (0.06)	0.05 (0.06)	**−0.15 (0.05)** *	−0.07 (0.07)	**−0.15 (0.06)** *
	Day 34	0.02 (0.05)	−0.03 (0.05)	0.06 (0.05)	**−0.14 (0.05)** *	−0.07 (0.05)	−0.08 (0.05)
Bleeding on probing	Day 14	−6% (4%)	1% (4%)	0% (4%)	−4% (4%)	−5% (4%)	−6% (4%)
	Day 28	−1% (4%)	−6% (5%)	7% (6%)	−2% (5%)	−5% (5%)	−1% (5%)
	Day 34	−9% (6%)	−2% (6%)	5% (6%)	−6% (6%)	**−13% (6%)** *	−3% (5%)

* Bold values indicate a result with *p* ≤ 0.05.

## 4 Discussion

The findings from the Phase 1/2a trial of KSL‐W indicate its potential use in humans as an anti‐plaque agent because it appears to prevent plaque formation and subsequent inflammation. Further, the absence of discernible differences in the frequency of AEs between the active treatment groups and the placebo groups suggests that it is safe in humans. Although the sample sizes in the Phase 1 dose groups were insufficient for robust statistical comparisons, the absence of trends indicating an association between increasing doses and more severe AEs is a positive indicator of safety as well.

The Phase 2a results bolster the proof of concept for KSL‐W as an effective intraoral antimicrobial agent. On comparing treatments against pooled placebo results, it was found that those who received gum with KSL‐W had less gingival inflammation and plaque accumulation after 2−4 weeks of active treatment, indicating a meaningful reduction of intraoral bacteria for study participants. Further analysis of results suggests that KSL‐W could be effective at 20 mg, but a 30 mg dose appears to minimize the side effects and maximize the antimicrobial impacts. Observed trends from Phase 2a consistently pointed toward improvements across outcome measures without any safety concerns as well. However, due to the limited size of each dosage group, more detailed comparisons could not be drawn, especially considering challenges in direct comparisons of changes from baseline between active and placebo treatments. This highlights the need for larger‐scale studies to strengthen the knowledge about KSL‐W and more accurately assess the efficacy suggested by our study results. Overall, these findings mark notable progress toward understanding the clinical potential of KSL‐W to address the problems associated with targeted intraoral microbial pharmacotherapy.

Results from both phases of this study provide strong justification for further research, with a potential Phase 2b exploring proof‐of‐concept endpoints further and Phase 3 serving as a pivotal study to definitively confirm the safety and verify the clinical efficacy of the product. Such future studies will need to establish the optimal dosage, frequency, and timing of use that will lead to a sustainable reduction in cariogenic bacteria for users. Though KSL‐W appears to be superior to currently available adjunctive products because of its selective activity against cariogenic bacteria, inferior substantivity is a potential challenge for daily use. Therefore, it will be critical for future studies to assess the quantitative changes in pathogenic and commensal bacteria to verify the selective activity against cariogenic bacteria found in in vitro studies.

The ultimate goal is to obtain FDA approval for the KSL‐W APCG as an OTC product. This achievement would signify a significant advancement in providing oral healthcare because it would empower all healthcare providers—not only dentists and hygienists—to play a significant role in promoting oral health, especially in populations with limited access to dental care. OTC approval would enable almost anyone to dispense this gum as an adjunctive for those unable to carry out routine daily oral hygiene, representing a potentially crucial intervention in populations where the risk for poor oral health, especially dental caries, is elevated (Marsh 2010; Merchan and Ismail 2021).

This gum holds particular promise for persistently transient or displaced individuals who face challenges in maintaining consistent access to potable running water for daily oral care practices like brushing and flossing. The United Nations estimates that over 100 million people globally live in temporary shelters, and approximately 30 million individuals in the United States lack reliable access to potable water (Cushing, Dobbin, and Jelks 2023; USA for UNHCR 2024). Being formulated as a gum offers a practical solution because it is easy to store, transport, and dispense, enhancing compliance and making it adaptable to various circumstances. This stands in sharp contrast to our current best adjunctive methods, often cumbersome liquids that not only pose logistical challenges but also indiscriminately eliminate commensal oral biota, contributing to oral dysbiosis and exacerbating oral health issues in these vulnerable populations (Syed and Shrivastava 2016; Rosier, Marsh, and Mira 2018).

Chewing gum formulated with KSL‐W could also potentially help sustain the oral health of individuals who are normally not at high risk for poor oral health but find themselves temporarily without access to care or the ability to adequately perform oral hygiene. One population with this problem is military service members deployed in austere environments where access to dental care is severely limited, proper oral hygiene is difficult to perform, and members routinely consume calorie‐dense food and high levels of caffeine, a stimulant known to cause xerostomia (dry mouth), a major risk factor for dental caries (Simecek and Diefenderfer 2010). Using this product would not only potentially help eliminate cariogenic bacteria, preserving beneficial oral flora and protecting the teeth from future insults, but also the act of chewing (mastication) stimulates salivary flow, which helps remove food debris from the mouth through swallowing and address stimulant‐induced xerostomia.

## 5 Conclusion

The Phase 1/2a trial provides initial safety data of KSL‐W use in humans and supports the antibacterial properties observed in vivo. The successful development of this gum holds the promise of substantially enhancing oral health across diverse populations. However, further studies are imperative to conclusively determine the effectiveness and safety of chewing gum containing KSL‐W, especially considering the small sample size of this investigation. The results from this trial serve as a promising foundation, underscoring the need for continued research and rigorous investigation to unlock the full potential of KSL‐W in promoting oral health and realizing its positive impact on a broader scale.

## Author Contributions

J.B.R. conducted the literature review, performed the secondary data analysis, and wrote the manuscript. B.J.K. was part of the design and execution of the project, collecting and organizing data, as well as editing the manuscript. K.P.L. was part of the design and execution of the project; he provided expert consultation during data analysis and editing of the manuscript. J.B.R. and B.J.K. contributed equally to the final manuscript.

## Ethics Statement

This study adheres to ethical standards and received approval from relevant institutional review boards. The manuscript was approved for publication by the authors' respective institutions.

## Consent

Informed consent was obtained from all participants.

## Conflicts of Interest

The authors declare no conflicts of interest.

## Supporting information

Supporting information.

Supporting information.

## Data Availability

The data from our Phase 1/2a clinical trial are available upon request. Interested researchers can contact the corresponding author for details.
