# Supplementary material for: Combined Phase 1/2a Initial Clinical Safety Trials and Proof‐of‐Concept Assessment of a Novel Antimicrobial Peptide KSL‐W Anti‐Plaque Chewing Gum
Source: Clin Exp Dent Res. 2024 Oct 28;10(6):e943. doi: 10.1002/cre2.943 (PMC11516077; doi:10.1002/cre2.943)
Supplement: Supplementary file 1 — Supporting information. [file CRE2-10-e943-s002.docx]

## **Supplemental Table 1: Adverse Event Summary for Phase 1**

| SOC and Preferred Term | Dose-Group 3a | | Dose-Group 4a | | Dose-Group 5a | | Dose-Group 8a | | Dose-Group 9a | |
| --- | --- | --- | --- | --- | --- | --- | --- | --- | --- | --- |
|  | 6 mg (N=4) | Placebo (N=2) | 10 mg (N=4) | Placebo (N=2) | 20 mg (N=7) | Placebo (N=3) | 75 mg (N=7) | Placebo (N=3) | 100 mg (N=7) | Placebo (N=3) |
| Gastrointestinal disorders | | | | | | | | | | |
| Abdominal discomfort |  |  |  |  | 1 |  |  |  |  |  |
| Dyspepsia |  |  |  |  |  |  | 1 |  |  |  |
| Mouth swelling |  |  |  |  |  |  | 1 |  |  |  |
| Mouth ulceration |  | 1 |  |  |  |  |  |  |  |  |
| Oral mucosal erythema |  |  |  |  |  | 1 |  |  |  |  |
| Injury, poisoning and procedural complications | | | | | | | | | | |
| Muscle strain |  |  |  | 1 |  |  |  |  |  |  |
| Investigations | | | | | | | | | | |
| Blood pressure increased |  |  |  |  |  |  |  |  | 1 |  |
| Musculoskeletal and connective tissue disorders | | | | | | | | | | |
| Musculoskeletal stiffness |  |  |  |  |  | 1 |  |  |  |  |
| Nervous system disorders | | | | | | | | | | |
| Headache |  |  |  |  |  |  |  |  | 1 |  |
| Respiratory, thoracic and mediastinal disorders | | | | | | | | | | |
| Throat irritation |  |  |  |  |  |  | 1 |  |  |  |
| Groups with no adverse events are not listed. | | | | | | | | | | |

## **Supplemental Table 2: Adverse Event Summary for Phase 2a**

| SOC and Preferred Term | Dose-Group 1b | | Dose-Group 2b | | Dose-Group 3b | | Dose-Group 4b | | Dose-Group 5b | | Dose-Group 6b | | Dose-Group 7b | |
| --- | --- | --- | --- | --- | --- | --- | --- | --- | --- | --- | --- | --- | --- | --- |
|  | 4mg (N=4) | Placebo (N=2) | 6mg (N=7) | Placebo (N=3) | 10mg (N=7) | Placebo (N=2) | 20mg (N=6) | Placebo (N=3) | 30mg (N=7) | Placebo (N=3) | 50mg (N=7) | Placebo (N=3) | 75mg (N=7) | Placebo (N=3) |
| Ear and labyrinth disorders | | | | | | | | | | | | | | |
| Vertigo |  |  |  |  |  |  |  |  |  | 1 |  |  |  |  |
| Gastrointestinal disorders | | | | | | | | | | | | | | |
| Abdominal discomfort |  |  |  |  | 1 |  |  |  |  | 1 |  |  | 1 |  |
| Abdominal distension |  |  |  |  |  |  |  | 1 |  |  |  |  |  |  |
| Coating in mouth |  |  |  |  |  |  |  |  |  |  |  |  | 1 |  |
| Dyspepsia |  |  |  |  |  |  | 1 |  |  |  |  |  |  |  |
| Flatulence |  |  | 1 |  |  |  |  |  |  |  |  |  |  |  |
| Food poisoning |  |  |  |  |  |  |  |  | 1 |  | 1 |  |  |  |
| Gingival ulceration |  |  |  |  |  |  |  |  | 1 |  |  |  |  |  |
| Mouth haemorrhage |  |  |  |  |  |  |  |  | 1 |  |  |  |  |  |
| Mouth ulceration |  | 1 |  |  | 1 |  |  |  |  | 1 |  |  |  |  |
| Oral mucosal erythema |  |  |  |  | 1 |  |  |  | 1 |  |  |  |  |  |
| Oral pain |  |  |  |  |  |  |  |  | 1 |  |  |  |  |  |
| Tongue coated |  |  |  |  |  |  |  |  |  |  | 3 |  |  |  |
| Tongue pigmentation |  |  |  |  |  |  |  |  |  |  |  |  | 1 |  |
| Tooth discolouration |  |  |  |  |  |  |  |  |  |  | 2 |  | 2 |  |
| General disorders and administration site conditions | | | | | | | | | | | | | | |
| Device connection issue |  |  |  |  |  |  |  | 1 |  |  |  |  |  |  |
| Infections and infestations | | | | | | | | | | | | | | |
| Ear infection |  |  | 1 |  |  |  |  |  |  |  |  |  |  |  |
| Nasopharyngitis |  |  | 1 | 1 | 1 |  |  |  |  |  |  |  | 3 |  |
| Sinusitis |  |  |  |  |  |  |  |  | 1 |  |  |  |  |  |
| Injury, poisoning and procedural complications | | | | | | | | | | | | | | |
| Arthropod bite |  |  |  |  |  |  |  |  |  |  | 1 |  |  |  |
| Mouth injury |  | 1 |  |  |  |  |  |  | 1 |  |  |  |  |  |
| Muscle strain | 1 |  |  |  |  |  |  |  |  |  |  |  |  |  |
| Thermal burn | 1 |  |  |  |  |  |  |  |  |  |  |  |  |  |
| Investigations | | | | | | | | | | | | | | |
| Aspartate aminotransferase increased |  | 1 |  |  |  |  |  |  |  |  |  |  |  |  |
| Blood glucose increased | 1 |  |  |  |  |  |  |  |  |  |  |  |  |  |
| Glucose urine present | 1 |  |  |  |  |  |  |  |  |  |  |  |  |  |
| Nervous system disorders | | | | | | | | | | | | | | |
| Ageusia |  |  |  |  |  |  |  |  |  |  |  |  | 1 |  |
| Dysgeusia |  |  |  |  |  |  |  |  |  |  | 2 |  | 3 |  |
| Headache |  |  |  |  |  |  |  |  | 1 | 1 |  | 1 |  |  |
| Respiratory, thoracic and mediastinal disorders | | | | | | | | | | | | | | |
| Oropharyngeal pain |  |  |  |  |  |  |  |  | 1 |  |  |  |  |  |
| Throat tightness |  |  |  |  |  |  |  |  |  |  |  |  | 1 |  |

## **Supplemental Figure 1: Summary of Subject Disposition for Phase 1**

## **Supplemental Figure 2: Summary of Subject Disposition for Phase 2a**
