# Supplementary material for: Combined Phase 1/2a Initial Clinical Safety Trials and Proof‐of‐Concept Assessment of a Novel Antimicrobial Peptide KSL‐W Anti‐Plaque Chewing Gum
Source: Clin Exp Dent Res. 2024 Oct 28;10(6):e943. doi: 10.1002/cre2.943 (PMC11516077; doi:10.1002/cre2.943)
Supplement: Supplementary file 2 — Supporting information. [file CRE2-10-e943-s001.docx]

Supplemental Table 3: Whole Mouth Plaque Index Scores for Phase 2a

|  | | | | Dose-Group 1b | | Dose-Group 2b | | Dose-Group 3b | | Dose-Group 4b | | Dose-Group 5b | | Dose-Group 6b | | Dose-Group 7b | |
| --- | --- | --- | --- | --- | --- | --- | --- | --- | --- | --- | --- | --- | --- | --- | --- | --- | --- |
|  |  |  |  | 4mg (N=4) | Placebo (N=2) | 6mg (N=7) | Placebo (N=3) | 10mg (N=7) | Placebo (N=2) | 20mg (N=6) | Placebo (N=3) | 30mg (N=7) | Placebo (N=3) | 50mg (N=7) | Placebo (N=3) | 75mg (N=7) | Placebo (N=3) |
|  |  | Overall | n | 4 | 2 | 7 | 3 | 7 | 2 | 6 | 3 | 7 | 3 | 7 | 3 | 7 | 3 |
|  |  |  | Mean | 3.78 | 4.07 | 3.83 | 3.88 | 3.77 | 4.00 | 4.15 | 4.19 | 4.12 | 4.14 | 4.06 | 4.14 | 2.95 | 2.58 |
|  |  |  | Median | 3.96 | 4.07 | 3.79 | 3.88 | 3.83 | 4.00 | 4.15 | 4.16 | 4.14 | 4.11 | 4.09 | 4.18 | 2.90 | 2.56 |
|  |  |  | SD | 0.58 | 0.20 | 0.24 | 0.22 | 0.58 | 0.10 | 0.09 | 0.16 | 0.17 | 0.11 | 0.22 | 0.24 | 0.25 | 0.23 |
|  |  |  | Min | 2.98 | 3.93 | 3.53 | 3.65 | 2.77 | 3.93 | 4.05 | 4.04 | 3.83 | 4.06 | 3.80 | 3.89 | 2.62 | 2.37 |
|  |  |  | Max | 4.22 | 4.21 | 4.16 | 4.10 | 4.40 | 4.07 | 4.26 | 4.35 | 4.38 | 4.26 | 4.40 | 4.35 | 3.31 | 2.82 |
|  |  | Day 0 | n | 4 | 2 | 7 | 3 | 7 | 2 | 6 | 3 | 7 | 3 | 7 | 3 | 7 | 3 |
|  |  |  | Mean | 3.80 | 4.27 | 3.95 | 4.12 | 3.77 | 4.12 | 4.08 | 4.18 | 3.98 | 4.11 | 3.89 | 4.14 | 3.74 | 3.41 |
|  |  |  | Median | 4.02 | 4.27 | 3.99 | 4.05 | 3.80 | 4.12 | 4.02 | 4.18 | 4.01 | 4.13 | 3.95 | 4.23 | 3.91 | 3.44 |
|  |  |  | SD | 0.58 | 0.15 | 0.27 | 0.16 | 0.32 | 0.05 | 0.14 | 0.08 | 0.12 | 0.04 | 0.24 | 0.18 | 0.37 | 0.07 |
|  |  |  | Min | 2.95 | 4.17 | 3.59 | 4.02 | 3.13 | 4.08 | 3.98 | 4.10 | 3.76 | 4.06 | 3.57 | 3.94 | 3.07 | 3.33 |
|  |  |  | Max | 4.21 | 4.38 | 4.27 | 4.30 | 4.11 | 4.15 | 4.36 | 4.26 | 4.15 | 4.13 | 4.23 | 4.26 | 4.17 | 3.46 |
|  |  | Day 14 | n | 4 | 2 | 7 | 3 | 7 | 2 | 5 | 3 | 6 | 3 | 7 | 3 | 7 | 3 |
|  |  |  | Mean | 4.00 | 4.21 | 3.83 | 4.02 | 3.78 | 4.16 | 3.94 | 4.19 | 3.66 | 3.97 | 3.37 | 4.21 | 3.21 | 3.69 |
|  |  |  | Median | 4.08 | 4.21 | 3.77 | 4.11 | 3.79 | 4.16 | 3.93 | 4.20 | 3.66 | 4.01 | 3.46 | 4.19 | 3.19 | 3.77 |
|  |  |  | SD | 0.28 | 0.13 | 0.21 | 0.23 | 0.27 | 0.15 | 0.14 | 0.19 | 0.21 | 0.12 | 0.36 | 0.14 | 0.21 | 0.19 |
|  |  |  | Min | 3.59 | 4.12 | 3.57 | 3.76 | 3.34 | 4.05 | 3.81 | 3.99 | 3.35 | 3.83 | 2.74 | 4.07 | 2.97 | 3.48 |
|  |  |  | Max | 4.23 | 4.30 | 4.13 | 4.20 | 4.19 | 4.27 | 4.16 | 4.37 | 3.96 | 4.05 | 3.85 | 4.36 | 3.61 | 3.83 |
|  |  | Day 28 | n | 4 | 2 | 7 | 3 | 6 | 2 | 5 | 3 | 6 | 3 | 6 | 3 | 7 | 2 |
|  |  |  | Mean | 4.17 | 4.16 | 3.76 | 4.15 | 3.90 | 4.21 | 3.82 | 4.20 | 3.57 | 4.06 | 3.44 | 4.19 | 3.15 | 3.60 |
|  |  |  | Median | 4.17 | 4.16 | 3.72 | 4.13 | 3.93 | 4.21 | 3.86 | 4.17 | 3.45 | 4.10 | 3.55 | 4.14 | 3.30 | 3.60 |
|  |  |  | SD | 0.18 | 0.22 | 0.20 | 0.09 | 0.18 | 0.15 | 0.10 | 0.12 | 0.32 | 0.07 | 0.42 | 0.16 | 0.35 | 0.03 |
|  |  |  | Min | 3.95 | 4.01 | 3.55 | 4.07 | 3.67 | 4.10 | 3.68 | 4.11 | 3.22 | 3.98 | 2.84 | 4.07 | 2.56 | 3.58 |
|  |  |  | Max | 4.39 | 4.32 | 4.05 | 4.25 | 4.10 | 4.32 | 3.90 | 4.33 | 4.06 | 4.10 | 3.95 | 4.38 | 3.50 | 3.63 |
|  |  | Day 34 | n | 4 | 2 | 7 | 3 | 6 | 2 | 5 | 3 | 5 | 3 | 6 | 3 | 7 | 2 |
|  |  |  | Mean | 4.02 | 4.27 | 3.78 | 3.97 | 3.97 | 4.11 | 3.94 | 4.18 | 3.76 | 4.07 | 3.68 | 3.97 | 3.54 | 3.88 |
|  |  |  | Median | 4.14 | 4.27 | 3.73 | 4.09 | 3.96 | 4.11 | 3.93 | 4.15 | 3.83 | 4.13 | 3.74 | 3.87 | 3.63 | 3.88 |
|  |  |  | SD | 0.34 | 0.08 | 0.19 | 0.28 | 0.15 | 0.00 | 0.05 | 0.18 | 0.18 | 0.14 | 0.39 | 0.22 | 0.31 | 0.24 |
|  |  |  | Min | 3.52 | 4.21 | 3.62 | 3.65 | 3.78 | 4.10 | 3.87 | 4.03 | 3.53 | 3.91 | 3.19 | 3.82 | 3.16 | 3.71 |
|  |  |  | Max | 4.29 | 4.33 | 4.15 | 4.15 | 4.23 | 4.11 | 3.99 | 4.38 | 3.93 | 4.17 | 4.28 | 4.23 | 3.90 | 4.05 |

Supplemental Table 4: Whole Mouth Gingival Index Scores for Phase 2a

|  | | | | Dose-Group 1b | | Dose-Group 2b | | Dose-Group 3b | | Dose-Group 4b | | Dose-Group 5b | | Dose-Group 6b | | Dose-Group 7b | |
| --- | --- | --- | --- | --- | --- | --- | --- | --- | --- | --- | --- | --- | --- | --- | --- | --- | --- |
|  |  |  |  | 4mg (N=4) | Placebo (N=2) | 6mg (N=7) | Placebo (N=3) | 10mg (N=7) | Placebo (N=2) | 20mg (N=6) | Placebo (N=3) | 30mg (N=7) | Placebo (N=3) | 50mg (N=7) | Placebo (N=3) | 75mg (N=7) | Placebo (N=3) |
|  |  | Overall | n | 4 | 2 | 7 | 3 | 7 | 2 | 6 | 3 | 7 | 3 | 7 | 3 | 7 | 3 |
|  |  |  | Mean | 2.66 | 2.78 | 2.51 | 2.70 | 2.58 | 2.58 | 2.63 | 2.64 | 2.55 | 2.50 | 2.31 | 2.33 | 2.43 | 2.16 |
|  |  |  | Median | 2.61 | 2.78 | 2.49 | 2.70 | 2.54 | 2.58 | 2.61 | 2.58 | 2.51 | 2.47 | 2.33 | 2.31 | 2.35 | 2.14 |
|  |  |  | SD | 0.20 | 0.17 | 0.10 | 0.05 | 0.14 | 0.02 | 0.16 | 0.17 | 0.08 | 0.09 | 0.06 | 0.11 | 0.35 | 0.30 |
|  |  |  | Min | 2.48 | 2.66 | 2.40 | 2.65 | 2.42 | 2.57 | 2.40 | 2.51 | 2.45 | 2.43 | 2.23 | 2.23 | 1.98 | 1.87 |
|  |  |  | Max | 2.95 | 2.90 | 2.68 | 2.76 | 2.84 | 2.60 | 2.86 | 2.83 | 2.65 | 2.61 | 2.42 | 2.45 | 3.02 | 2.48 |
|  |  | Day 0 | n | 4 | 2 | 7 | 3 | 7 | 2 | 6 | 3 | 7 | 3 | 7 | 3 | 7 | 3 |
|  |  |  | Mean | 2.61 | 2.73 | 2.66 | 2.64 | 2.56 | 2.57 | 2.57 | 2.59 | 2.55 | 2.57 | 2.31 | 2.35 | 2.32 | 2.16 |
|  |  |  | Median | 2.58 | 2.73 | 2.64 | 2.64 | 2.58 | 2.57 | 2.52 | 2.56 | 2.54 | 2.45 | 2.23 | 2.37 | 2.33 | 2.18 |
|  |  |  | SD | 0.17 | 0.17 | 0.07 | 0.05 | 0.11 | 0.10 | 0.18 | 0.10 | 0.08 | 0.22 | 0.15 | 0.12 | 0.15 | 0.12 |
|  |  |  | Min | 2.45 | 2.61 | 2.58 | 2.60 | 2.41 | 2.51 | 2.33 | 2.50 | 2.44 | 2.43 | 2.13 | 2.23 | 2.12 | 2.03 |
|  |  |  | Max | 2.83 | 2.85 | 2.80 | 2.69 | 2.69 | 2.64 | 2.86 | 2.70 | 2.65 | 2.83 | 2.53 | 2.46 | 2.52 | 2.26 |
|  |  | Day 14 | n | 4 | 2 | 7 | 3 | 7 | 2 | 5 | 3 | 6 | 3 | 7 | 3 | 7 | 3 |
|  |  |  | Mean | 2.58 | 2.68 | 2.66 | 2.78 | 2.60 | 2.68 | 2.59 | 2.64 | 2.44 | 2.45 | 2.28 | 2.35 | 2.23 | 2.16 |
|  |  |  | Median | 2.53 | 2.68 | 2.66 | 2.78 | 2.59 | 2.68 | 2.55 | 2.58 | 2.42 | 2.40 | 2.29 | 2.29 | 2.21 | 2.14 |
|  |  |  | SD | 0.14 | 0.08 | 0.06 | 0.05 | 0.12 | 0.13 | 0.20 | 0.11 | 0.12 | 0.15 | 0.06 | 0.18 | 0.08 | 0.06 |
|  |  |  | Min | 2.48 | 2.63 | 2.61 | 2.73 | 2.45 | 2.58 | 2.30 | 2.57 | 2.31 | 2.33 | 2.19 | 2.21 | 2.14 | 2.11 |
|  |  |  | Max | 2.78 | 2.73 | 2.78 | 2.83 | 2.79 | 2.77 | 2.84 | 2.77 | 2.58 | 2.62 | 2.36 | 2.55 | 2.36 | 2.22 |
|  |  | Day 28 | n | 4 | 2 | 7 | 3 | 6 | 2 | 5 | 3 | 6 | 3 | 6 | 3 | 7 | 2 |
|  |  |  | Mean | 2.70 | 2.78 | 2.66 | 2.70 | 2.58 | 2.71 | 2.65 | 2.66 | 2.42 | 2.48 | 2.25 | 2.24 | 2.23 | 2.23 |
|  |  |  | Median | 2.72 | 2.78 | 2.69 | 2.67 | 2.57 | 2.71 | 2.60 | 2.65 | 2.42 | 2.44 | 2.25 | 2.22 | 2.21 | 2.23 |
|  |  |  | SD | 0.19 | 0.19 | 0.08 | 0.10 | 0.13 | 0.17 | 0.21 | 0.04 | 0.10 | 0.10 | 0.15 | 0.09 | 0.09 | 0.01 |
|  |  |  | Min | 2.46 | 2.64 | 2.49 | 2.62 | 2.44 | 2.60 | 2.36 | 2.63 | 2.30 | 2.41 | 2.07 | 2.16 | 2.13 | 2.22 |
|  |  |  | Max | 2.90 | 2.91 | 2.72 | 2.81 | 2.82 | 2.83 | 2.89 | 2.71 | 2.55 | 2.60 | 2.49 | 2.34 | 2.35 | 2.24 |
|  |  | Day 34 | n | 4 | 2 | 7 | 3 | 6 | 2 | 5 | 3 | 5 | 3 | 6 | 3 | 7 | 2 |
|  |  |  | Mean | 2.66 | 2.76 | 2.65 | 2.70 | 2.51 | 2.60 | 2.62 | 2.56 | 2.38 | 2.44 | 2.23 | 2.27 | 2.27 | 2.27 |
|  |  |  | Median | 2.65 | 2.76 | 2.70 | 2.72 | 2.53 | 2.60 | 2.61 | 2.48 | 2.38 | 2.40 | 2.21 | 2.28 | 2.23 | 2.27 |
|  |  |  | SD | 0.19 | 0.09 | 0.11 | 0.09 | 0.09 | 0.18 | 0.21 | 0.19 | 0.06 | 0.16 | 0.10 | 0.06 | 0.10 | 0.11 |
|  |  |  | Min | 2.49 | 2.69 | 2.51 | 2.60 | 2.35 | 2.47 | 2.36 | 2.42 | 2.30 | 2.30 | 2.12 | 2.21 | 2.18 | 2.20 |
|  |  |  | Max | 2.84 | 2.82 | 2.77 | 2.77 | 2.58 | 2.73 | 2.90 | 2.77 | 2.48 | 2.61 | 2.39 | 2.33 | 2.43 | 2.35 |

Supplemental Table 5: Whole Mouth Gingival Bleeding on Probing (BOP) Scores for Phase 2a

|  | | | | Dose-Group 1b | | Dose-Group 2b | | Dose-Group 3b | | Dose-Group 4b | | Dose-Group 5b | | Dose-Group 6b | | Dose-Group 7b | |
| --- | --- | --- | --- | --- | --- | --- | --- | --- | --- | --- | --- | --- | --- | --- | --- | --- | --- |
|  |  |  |  | 4mg (N=4) | Placebo (N=2) | 6mg (N=7) | Placebo (N=3) | 10mg (N=7) | Placebo (N=2) | 20mg (N=6) | Placebo (N=3) | 30mg (N=7) | Placebo (N=3) | 50mg (N=7) | Placebo (N=3) | 75mg (N=7) | Placebo (N=3) |
|  |  | Overall | n | 4 | 2 | 7 | 3 | 7 | 2 | 6 | 3 | 7 | 3 | 7 | 3 | 7 | 3 |
|  |  |  | Mean | 16% | 27% | 26% | 22% | 17% | 27% | 30% | 37% | 25% | 17% | 20% | 19% | 13% | 11% |
|  |  |  | Median | 10% | 27% | 29% | 14% | 9% | 27% | 32% | 35% | 23% | 18% | 17% | 18% | 10% | 8% |
|  |  |  | SD | 15% | 26% | 10% | 16% | 16% | 7% | 14% | 26% | 8% | 5% | 15% | 8% | 9% | 9% |
|  |  |  | Min | 5% | 8% | 10% | 11% | 5% | 23% | 7% | 11% | 15% | 12% | 5% | 11% | 4% | 4% |
|  |  |  | Max | 38% | 45% | 36% | 41% | 45% | 32% | 48% | 63% | 39% | 21% | 46% | 27% | 28% | 21% |
|  |  | Day 0 | n | 4 | 2 | 7 | 3 | 7 | 2 | 6 | 3 | 7 | 3 | 7 | 3 | 7 | 3 |
|  |  |  | Mean | 20% | 37% | 29% | 19% | 16% | 26% | 29% | 38% | 31% | 19% | 25% | 27% | 21% | 17% |
|  |  |  | Median | 16% | 37% | 30% | 17% | 11% | 26% | 30% | 32% | 30% | 21% | 21% | 21% | 17% | 16% |
|  |  |  | SD | 12% | 31% | 12% | 6% | 11% | 5% | 19% | 28% | 14% | 8% | 19% | 12% | 16% | 4% |
|  |  |  | Min | 11% | 15% | 11% | 15% | 5% | 23% | 5% | 13% | 14% | 10% | 7% | 20% | 4% | 13% |
|  |  |  | Max | 38% | 58% | 41% | 26% | 34% | 30% | 58% | 69% | 52% | 25% | 57% | 41% | 45% | 21% |
|  |  | Day 14 | n | 4 | 2 | 7 | 3 | 7 | 2 | 5 | 3 | 6 | 3 | 7 | 3 | 7 | 3 |
|  |  |  | Mean | 16% | 28% | 21% | 24% | 17% | 15% | 25% | 33% | 24% | 20% | 19% | 32% | 15% | 15% |
|  |  |  | Median | 11% | 28% | 20% | 19% | 11% | 15% | 22% | 33% | 22% | 12% | 20% | 32% | 17% | 17% |
|  |  |  | SD | 15% | 24% | 11% | 13% | 15% | 11% | 17% | 20% | 14% | 14% | 8% | 1% | 7% | 9% |
|  |  |  | Min | 4% | 11% | 7% | 15% | 4% | 7% | 9% | 13% | 10% | 12% | 7% | 30% | 4% | 5% |
|  |  |  | Max | 39% | 45% | 38% | 39% | 48% | 23% | 52% | 54% | 48% | 37% | 31% | 33% | 24% | 23% |
|  |  | Day 28 | n | 4 | 2 | 7 | 3 | 6 | 2 | 5 | 3 | 6 | 3 | 6 | 3 | 7 | 2 |
|  |  |  | Mean | 16% | 24% | 25% | 23% | 13% | 25% | 33% | 28% | 25% | 24% | 17% | 31% | 22% | 13% |
|  |  |  | Median | 8% | 24% | 22% | 16% | 11% | 25% | 34% | 29% | 24% | 23% | 11% | 31% | 23% | 13% |
|  |  |  | SD | 18% | 29% | 9% | 14% | 10% | 12% | 21% | 8% | 16% | 3% | 17% | 18% | 9% | 1% |
|  |  |  | Min | 5% | 4% | 13% | 14% | 3% | 16% | 11% | 20% | 3% | 21% | 5% | 12% | 8% | 13% |
|  |  |  | Max | 42% | 45% | 37% | 40% | 29% | 33% | 63% | 36% | 50% | 27% | 50% | 49% | 38% | 14% |
|  |  | Day 34 | n | 4 | 2 | 7 | 3 | 6 | 2 | 5 | 3 | 5 | 3 | 6 | 3 | 7 | 2 |
|  |  |  | Mean | 18% | 46% | 18% | 22% | 16% | 28% | 31% | 31% | 22% | 12% | 10% | 24% | 19% | 20% |
|  |  |  | Median | 11% | 46% | 17% | 17% | 13% | 28% | 40% | 43% | 18% | 13% | 8% | 29% | 23% | 20% |
|  |  |  | SD | 18% | 11% | 10% | 15% | 12% | 27% | 17% | 21% | 13% | 6% | 5% | 14% | 10% | 4% |
|  |  |  | Min | 6% | 38% | 6% | 9% | 7% | 10% | 9% | 7% | 4% | 5% | 7% | 9% | 6% | 17% |
|  |  |  | Max | 46% | 53% | 37% | 39% | 40% | 47% | 48% | 45% | 36% | 17% | 20% | 35% | 29% | 23% |
